# Novel Thrombolytic Drug Based on Thrombin Cleavable Microplasminogen Coupled to a Single‐Chain Antibody Specific for Activated GPIIb/IIIa

**DOI:** 10.1161/JAHA.116.004535

**Published:** 2017-02-03

**Authors:** Thomas Bonnard, Zachary Tennant, Be'Eri Niego, Ruchi Kanojia, Karen Alt, Shweta Jagdale, Lok Soon Law, Sheena Rigby, Robert Lindsay Medcalf, Karlheinz Peter, Christoph Eugen Hagemeyer

**Affiliations:** ^1^ NanoBiotechnology Laboratory Australian Centre for Blood Diseases Monash University Melbourne Australia; ^2^ Vascular Biotechnology Laboratory Baker IDI Heart and Diabetes Institute Melbourne Australia; ^3^ Molecular Neurotrauma and Haemostasis Laboratory Australian Centre for Blood Diseases Monash University Melbourne Australia; ^4^ Atherothrombosis and Vascular Biology Laboratory Baker IDI Heart and Diabetes Institute Melbourne Australia; ^5^ RMIT University Melbourne Australia

**Keywords:** glycoproteins, plasminogen, platelet, thrombin, thrombolysis, thrombosis, Thrombosis, Vascular Disease, Coronary Artery Disease

## Abstract

**Background:**

Thrombolytic therapy for acute thrombosis is limited by life‐threatening side effects such as major bleeding and neurotoxicity. New treatment options with enhanced fibrinolytic potential are therefore required. Here, we report the development of a new thrombolytic molecule that exploits key features of thrombosis. We designed a recombinant microplasminogen modified to be activated by the prothrombotic serine‐protease thrombin (HtPlg), fused to an activation‐specific anti–glycoprotein IIb/IIIa single‐chain antibody (SCE5), thereby hijacking the coagulation system to initiate thrombolysis.

**Methods and Results:**

The resulting fusion protein named SCE5‐HtPlg shows in vitro targeting towards the highly abundant activated form of the fibrinogen receptor glycoprotein IIb/IIIa expressed on activated human platelets. Following thrombin formation, SCE5‐HtPlg is activated to contain active microplasmin. We evaluate the effectiveness of our targeted thrombolytic construct in two models of thromboembolic disease. Administration of SCE5‐HtPlg (4 μg/g body weight) resulted in effective thrombolysis 20 minutes after injection in a ferric chloride–induced model of mesenteric thrombosis (48±3% versus 92±5% for saline control, *P*<0.01) and also reduced emboli formation in a model of pulmonary embolism (*P*<0.01 versus saline). Furthermore, at these effective therapeutic doses, the SCE5‐HtPlg did not prolong bleeding time compared with saline (*P*=0.99).

**Conclusions:**

Our novel fusion molecule is a potent and effective treatment for thrombosis that enables in vivo thrombolysis without bleeding time prolongation. The activation of this construct by thrombin generated within the clot itself rather than by a plasminogen activator, which needs to be delivered systemically, provides a novel targeted approach to improve thrombolysis.

## Introduction

Thrombotic diseases such as acute myocardial infarction, ischemic stroke, and pulmonary embolism remain leading causes of death and disability.^1^ Fibrinolytic therapy with plasminogen activators has been proven to be beneficial and is widely used in the acute setting of thrombosis.^2^, ^3^, ^4^ However, in stroke, their benefit is restricted to a window of 4.5 hours and the dose administered is limited by damage to the central nervous system and lysis of homeostatic clots leading to fatal bleeding complications.^5^, ^6^

Thrombin is a key enzyme of the blood coagulation cascade as it activates platelets, catalyzes the polymerization of fibrinogen into fibrin, and converts factors V, VIII, XI, and XIII into their activated form.^7^ Its abundant generation from the prothrombinase complex, often referred to as the “thrombin burst,” is localized on the surface of activated platelets and is specific to thrombus sites.^8^ The central role of this serine protease has driven the development of several thrombin responsive clot‐lysing drugs. Potent fibrinolytic agents were synthesized from thrombin‐activatable prourokinase fused to single‐chain antibody targeting red blood cells or platelets and provided sustained thromboprophylaxis in vivo in mouse models.^9^, ^10^ Our group recently developed promising layer‐by‐layer nanocapsules that release urokinase upon degradation by thrombin.^11^ Another approach consisted of engineering a variant of human plasminogen to be cleaved into plasmin by thrombin.^12^ This thrombin‐cleavable plasminogen had promising outcomes in preclinical studies, which led to clinical trials.^13^, ^14^, ^15^ Unfortunately, the effective doses in the dose‐escalation trials induced significant bleeding complications.^16^

To reduce the bleeding complication associated with fibrinolytic agents and to enhance their therapeutic efficiency, new treatments have been developed with targeting moieties directed toward thrombus components in order to selectively concentrate the activity of the drug at the site of thrombus.^17^, ^18^, ^19^, ^20^, ^21^ Activated platelets are a main component of human thrombi, and integrin glycoprotein (GP)IIb/IIIa is the most abundant membrane protein expressed upon activation (≈80 000 receptors per platelet).^22^ Hence, integrin GPIIb/IIIa constitutes an attractive target for the development of clot‐specific thrombolytic drugs. Our group recently developed a new fibrinolytic agent by the fusion of single‐chain urokinase plasminogen activator to a small recombinant antibody (scFv_SCE5_) that targets the activated form of the platelet‐integrin GPIIb/IIIa.^23^ In that study, the targeting property allowed a substantial 6‐fold reduction in the therapeutic dosage that significantly reduced hemorrhagic risk.

Herein, we have combined both promising features of the previously developed thrombolytic agents (targeting and thrombin activatable plasminogen) into one fusion molecule. Furthermore, we utilized microplasmin, a truncated form of plasmin that lacks the 5 Kringle domains of full‐length plasminogen. The absence of the Kringle domains has several advantages: the inhibition rate of microplasmin by α_2_‐antiplasmin is reduced to 0.01% of the inhibition rate of intact plasmin, which makes it suitable for use as an intravenous therapeutic agent.^24^ In preclinical studies, microplasmin reduced ischemic brain damage, showed nonlysis‐dependent neuroprotective effects improving behavioral rating scores, and lower bleeding tendency at equally effective doses of tissue plasminogen activator (tPA).^25^, ^26^ Moreover, the smaller size of the entire fusion construct favors a better penetration within the core of blood clots. By using genetic engineering and cloning techniques, we replaced the plasminogen activator recognition loop (CPGRVVGGC) of human microplasminogen with the amino acid sequence of the thrombin recognition loop from Factor XI (CTTKIKPRIVGGC) and we fused this to an activation‐specific anti–GPIIb/IIIa single‐chain antibody (SCE5). We describe the production and in vitro and in vivo testing of this new clot‐specific thrombin‐cleavable human microplasminogen (HtPlg‐SCE5). Efficient thrombolytic capacities are measured in two different mouse models of thrombosis at a dose associated with no bleeding time prolongation. This novel fibrinolytic agent represents a promising alternative of plasminogen activators for thrombolysis therapy.

## Materials and Methods

### Generation, Expression, and Purification of Single‐Chain Antibodies Fused With Human Thrombin‐Activatable Plasminogen

The DNA sequence coding for the human thrombin‐activatable microplasminogen (HtPlg) was obtained from GeneArt (ThermoFisher Scientific, Waltham, MA). The HtPlg construct was then fused with two different single‐chain antibodies, the activation‐specific GPIIb/IIIa–targeted (SCE5) and –nontargeted (Mut‐scFv), as previously described.^27^, ^28^ The fusion constructs SCE5‐HtPlg and Mut‐scFv‐HtPlg were transfected in human embryonic kidney cells (freeStyleHEK 293‐Fcells; Life Technologies, Carlsbad, CA), suspension cells for production of the proteins, which were isolated by fast liquid protein chromatography with a nickel‐based metal affinity column Ni‐NTA (Invitrogen, Carlsbad, CA). The detailed procedures are available in the supplementary material.

### Cleavage of the HtPlg Proteins Into Microplasmin

The cleavage of SCE5‐HtPlg and Mut‐scFv‐HtPlg from thrombin incubation into microplasmin was studied in vitro with Western blot analysis and by spectrophotometry using the S2251 amidolytic assay. The detailed procedures are available the supplementary material.

### 96‐Well Plate Fibrinolysis Assay

All experiments involving blood samples collected from human volunteers were approved by The Alfred Hospital ethics committee (project 67/15). Written informed consent was obtained from all donors prior to phlebotomy. Blood was collected in sodium citrate 3.8% (w/v). Thrombi were formed in halo shape at the bottom of 96‐well plates with human blood collected from healthy volunteers. The degradation of the halo thrombi was measured with a plate reader (EnSpire Multimode; PerkinElmer, Waltham, MA) at 510 nm from the absorbance of the released blood in the solution as the thrombi progressively lyses the center of the well. Different concentration of plasmin, urokinase, SCE5‐HtPlg, or Mut‐scFv‐HtPlg (0.1 and 0.2 mg/mL) were tested (n=4). The detailed procedures are available in the supplementary material.

### Flow Cytometry

The affinity of the fusion proteins to GPIIb/IIIa expressed on human platelets was assessed by flow cytometry. Three samples of human platelet‐rich plasma (PRP) were prepared from human blood: nonactivated platelets (PRP), ADP‐activated platelets (PRP+ADP), and ADP‐activated and GPIIb/IIIa–blocked platelets (PRP+ADP+abciximab). Interaction of the Mut‐scFv‐HtPlg and SCE5‐HtPlg constructs labeled with fluorescein isothiocyanate (FITC) secondary antibody was assessed on a FACSCanto II Flow cytometer (BD Biosciences, Franklin Lakes, NJ). The detailed procedures are available in the supplementary material.

### Template Tail Bleeding, Hemoglobin, Albumin, and Plasma Fibrinogen Measurements

All experiments involving animals were approved by the Alfred Medical Research and Education Precinct Animal Ethics Committee (E/1534/2015/B and E/1589/2015/B). Tail bleeding times were determined using the template method^29^ after intravenous injection of several groups of drug: urokinase at 100 and 500 U/g body weight (BW); SCE5‐HtPlg at 2, 4, 8 μg/g BW; Mut‐scFv‐HtPlg at 2, 4, 8 μg/g BW; and saline (n=3). Hemoglobin and albumin in brain and gut as well as plasma fibrinogen levels were measured 24 hours after administration of urokinase at 500 U/g BW, SCE5‐HtPlg at 4 μg/g BW, and saline (n=3). The detailed procedures are available in the supplementary material.

### Endothelial Cells Permeability Assay

Permeability measurement of brain endothelial cells after various treatments was adapted from a previously described cell permeability assay in an in vitro model of the blood‐brain barrier.^30^ Detailed procedures are available in the supplementary material.

### Ferric Chloride–Induced Thrombosis in Mesenteric Vessels

Targeting and thrombolytic capacities of the HtPlg fusion proteins were tested in a mouse model of thrombosis induced by ferric chloride superfusion in mesenteric vessels performed as previously described.^31^ The detailed procedures are available in the supplementary material.

### Lung Embolism Model

Emboli were induced and fluorescently stained by intravenous injection (5 μL/g BW) of a mixture of Innovin and near‐infrared dye–labeled fibrinogen. Ten minutes after the induction of the prothrombotic mixture, 4 drug groups were intravenously injected: urokinase at 500 U/g BW, SCE5‐HtPlg at 4 μg/g BW, Mut‐scFv‐HtPlg at 4 μg/g BW, and saline (n=3). The number of emboli were measured via fibrinogen fluorescence within the lung harvested 50 minutes after treatment. The detailed procedures are available in the supplementary material.

### Statistical Analysis

All results are expressed as mean±SEM. Statistical analysis was performed with GraphPad Prism V6 (GraphPad Software, San Diego, CA). Multiple groups (Flow cytometry, tail bleeding, fibrinogen level in plasma, hemoglobin and albumin levels in brain and intestine, permeability level, each time point separately for thrombus degradation values in the ferric chloride–induced thrombosis model, and fibrinogen fluorescence in the pulmonary embolism model) were compared with 1‐way ANOVA and Tukey post‐tests. Parameters from in vitro fibrinolysis assay of SCE5‐HtPlg and Mut‐scFv‐HtPlg groups were compared with unpaired *t* tests. A difference of *P*<0.05 was considered significant.

## Results

### Production of Fusion Proteins SCE5‐HtPlg and Mut‐scFv‐HtPlg

The HtPlg was subcloned with the GPIIb/IIIa–targeted (SCE5) or the nontargeted (Mut‐scFv) single‐chain antibody (scFv) into the pSecTag vector system. The DNA amplification and restriction digest of the obtained SCE5‐HtPlg and Mut‐scFv‐HtPlg fragments were analyzed by gel electrophoresis (Figure S1A). After amplification with polymerase chain reaction (PCR) and restriction digest, the subcloned DNA of the SCE5‐HtPlg and the Mut‐scFv‐HtPlg were visualized at 1.8 kbp, which is the expected size since the digested HtPlg construct migrates at 0.8 kbp and the uncut pSecTag vector containing the scFvs migrates at 1 kbp. The sequences of both fusion constructs, represented in the pSecTag vector map (Figure S1A), were confirmed via DNA sequencing. The DNA of the SCE5‐HtPlg and the Mut‐scFv‐HtPlg was then transfected into HEK293 cells for production of the fusion proteins, which were isolated at around 75 and 55 kDa as shown on sodium dodecyl sulfate SDS‐PAGE and Western blot anti‐His analysis (Figure S1B).

### In Vitro Evaluation of the Conversion Into Microplasmin and of Thrombolytic Capacities

**Figure 1:**
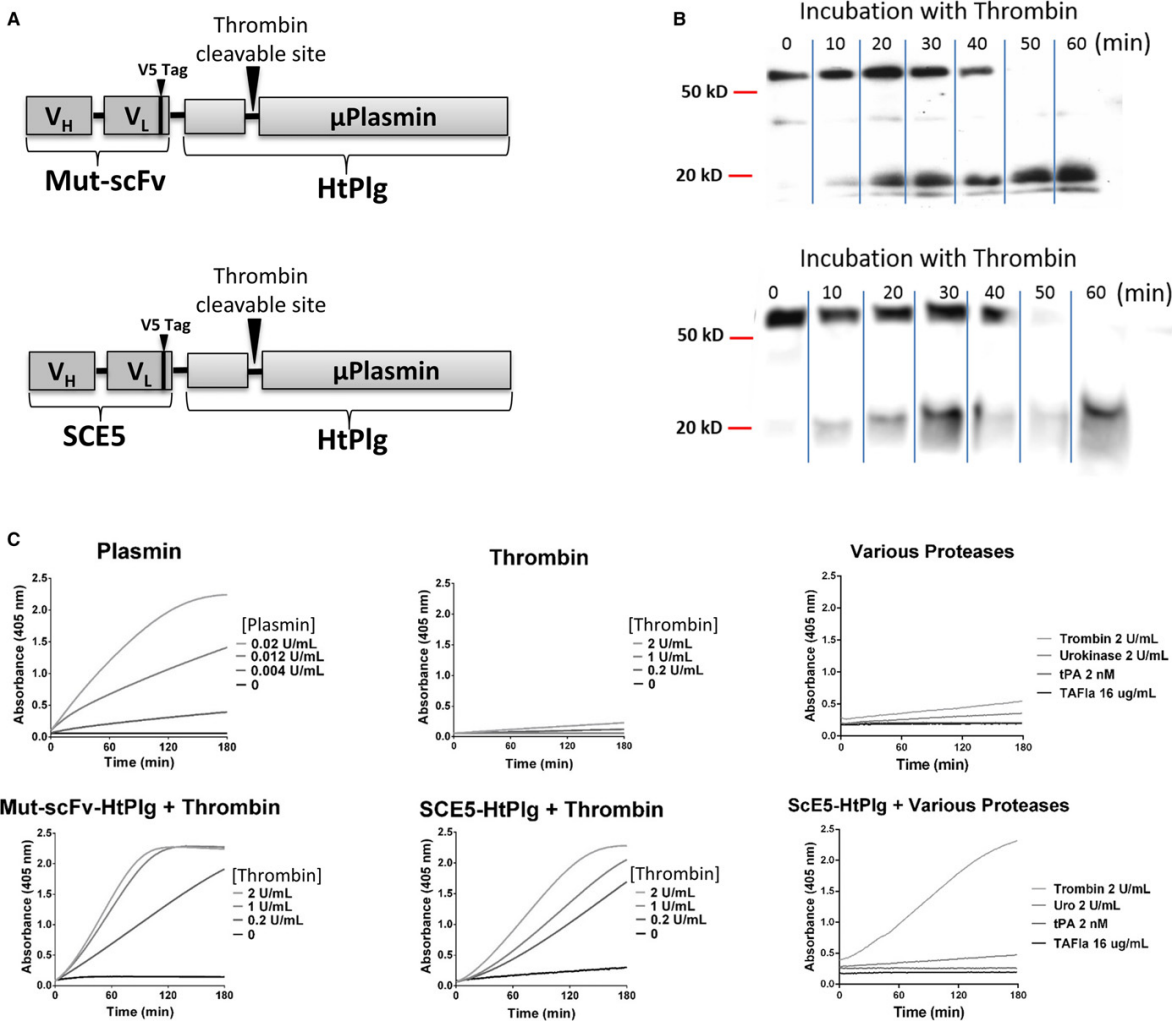
A, Schematic representation of the anti–glycoprotein (GP)IIb/IIIa single‐chain antibody (SCE5)–human thrombin‐activatable microplasminogen (HtPlg) and nontargeted control scFv HtPlg (Mut‐scFv‐HtPlg) constructs. The amino acid sequence of the plasminogen activator site from human plasminogen was substituted for the thrombin cleavage site from factor XIII. The HtPlg construct was then fused with two different single‐chain antibodies, one targeting activated GPIIb/IIIa (SCE5) and the other Mut‐scFv. B, Cleavage and generation of microplasmin after thrombin incubation was demonstrated in vitro. The Mut‐scFv‐HtPlg or the SCE5‐HtPlg (200 μg/mL) was incubated at 37°C with thrombin (3 U/mL) and samples were withdrawn at 0, 10, 20, 30, 40, 50, and 60 minutes, mixed with dithiothreitol and analyzed on Western blots using horseradish peroxidase coupled to an anti‐V5 antibody. C, The activation of the SCE5‐HtPlg (13 μg/mL) and of the Mut‐scFv‐HtPlg (13 μg/mL) to microplasmin after incubation with different thrombin concentrations (0, 0.2, 1, and 2 U/mL) was demonstrated. The SCE5‐HtPlg was additionally tested with urokinase (2 U/mL), tPA (2 nmol/L), and thrombin‐activatable fibrinolysis inhibitor (TAFIa) (16 μg/mL). Microplasmin generation was monitored over 2 hours by spectrophotometry at 405 nm with the plasmin chromogenic substrate S2251 (350 μmol/L). Positive control was obtained with different plasmin concentrations (0, 0.004, 0.012, and 0.02 U/mL) and negative control was obtained with only thrombin (0, 0.2, 1, and 2 U/mL).

**Figure 2:**
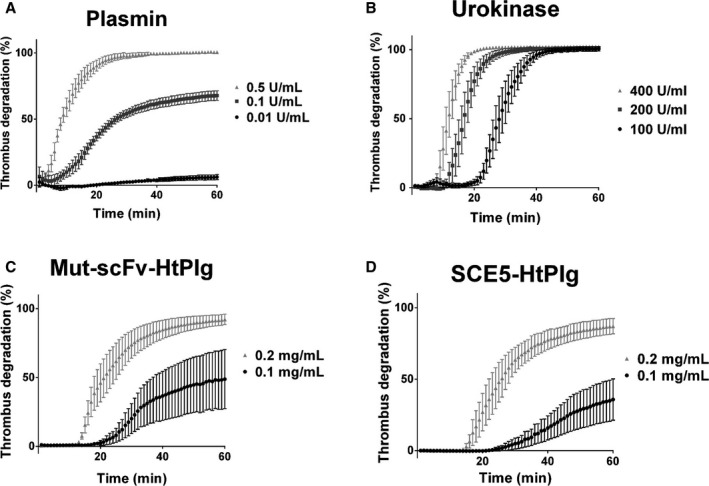
Fibrinolytic capacities of the anti–glycoprotein IIb/IIIa single‐chain antibody–human thrombin‐activatable microplasminogen (SCE5‐HtPlg) and nontargeted control scFv HtPlg (Mut‐scFv‐HtPlg) were tested in vitro on thrombi formed in a halo shape at the bottom of 96‐well plates. The degradation of the thrombi was monitored over 1 hour at 37°C by spectrophotometry from the absorbance of the blood progressively covering the center of the well. Fibrinolysis rates were determined using known activities of plasmin (A), urokinase (B) or different concentrations of Mut‐scFv‐HtPlg (C), and SCE5‐HtPlg (D). For each assay, positive control of the assay contained blood topped up to the final volume with buffer while the negative control contained a preprepared halo aggregate topped up with buffer to the final volume.

Western blot analysis revealed that both constructs at 200 μg/mL were fully cleaved over 1 hour when incubated at 37°C with 3 U/mL thrombin (Figure 1B). At t=0, only the full constructs are revealed by the anti‐V5 antibody. From 20 to 40 minutes incubation, a certain amount of the constructs are cleaved into microplasmin and a portion that contains the single‐chain antibodies and the V5 tag. From 40 minutes incubation, the whole constructs are fully cleaved. To investigate the effect of thrombin at inducing the cleavage of the SCE5‐HtPlg and Mut‐scFv‐HtPlg into microplasmin, the fusion proteins were exposed to simulate thrombotic conditions with different thrombin concentrations (0, 0.2, 1, and 2 U/mL), and the generation of microplasmin was monitored over 2 hours by spectrophotometry using the S2251 amidolytic assay (Figure 1C). The thrombin concentration–dependent kinetics of the SCE5‐HtPlg and the Mut‐scFv‐HtPlg compared with the low signal obtained without SCE5‐HtPlg verify the thrombin‐specific activation feature of the drug. On the other hand, the addition of urokinase, tPA, or thrombin‐activatable fibrinolysis inhibitor (TAFIa), within the similar activity range as the high thrombin dose tested, did not trigger any generation of microplasmin when mixed with the SCE5‐HtPlg. The capacities of the SCE5‐HtPlg and the Mut‐scFv‐HtPlg to lyse whole blood thrombi were assessed in vitro and compared with the fibrinolysis obtained with human plasmin and urokinase. The addition of human plasmin resulted in a direct initiation of fibrinolysis at a rate increasing with the concentration of plasmin (Figure 2A). At 0.5 U/mL, a full degradation (over 95%) was obtained after 24±3 minutes; at 0.1 U/mL, the degradation was limited to 68±3% degradation; and at 0.01 U/mL, almost no degradation was observed. The addition of urokinase resulted in a different degradation profile (Figure 2B). A short delay period was observed before the initiation of the degradation. This initiation time decreased with the concentration of urokinase: 21±2 with 100 U/mL, 13±1 minutes with 200 U/mL, and 9±1 minutes with 400 U/mL. However, all urokinase concentrations resulted in full degradation. The Mut‐scFv‐HtPlg and the SCE5‐HtPlg (Figure 2C and 2D) resulted in degradation profiles combining the plateau effect observed with plasmin and the initiation time effect observed with urokinase. With the addition of Mut‐scFv‐HtPlg or SCE5‐HtPlg, maximal degradation of 36±11% and 49±17%, respectively, at 0.1 mg/mL (*P*=0.51) and 87±4% and 92±3%, respectively, at 0.2 mg/mL (*P*=0.58) were reached. Initiation times of 46±15 and 30±6 minutes, respectively, at 0.1 mg/mL (*P*=0.17) and 17±1 and 14±1 minutes, respectively, at 0.2 mg/mL (*P*=0.87) were measured. The addition of higher concentrations (0.3 and 0.4 mg/mL) of Mut‐scFv‐HtPlg and SCE5‐HtPlg did not shorten the initiation time (data not shown).

We repeated this in vitro thrombolysis study with urokinase and SCE5‐HtPlg in the presence of exogenous plasminogen activator inhibitor‐1 (PAI‐1) and TAFIa (Figure S2). The thrombolysis initiation from urokinase was delayed by both PAI‐1 and TAFIa (38±3 for urokinase 200 U/mL+PAI‐1 6 nmol/L and 36±7 for urokinase 200 U/mL+TAFIa 20 nmol/L versus 25±2 for urokinase 200 U/mL only, *P*<0.05), whereas it was stable with SCE5‐HtPlg at 0.2 mg/mL.

### Assessment of Bleeding Consequences

**Figure 3:**
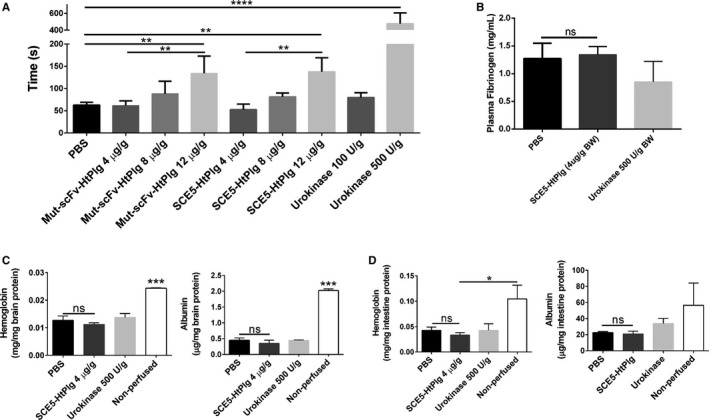
A, Template tail bleeding times were used to assess the hemostatic impact of the different constructs in mice treated with PBS, nontargeted control scFv HtPlg (Mut‐scFv‐HtPlg; 4, 8, and 12 μg/g body weight [BW]), anti–glycoprotein IIb/IIIa single‐chain antibody–human thrombin‐activatable microplasminogen (SCE5‐HtPlg; 4, 8, and 12 μg/g BW), and urokinase (100 and 500 U/g BW). Bleeding time was recorded between the section and the arrest of bleeding. B, Fibrinogen levels were measured in mice treated with urokinase (500 U/g BW), SCE5‐HtPlg (4 μg/g BW), and saline 24 hours after treatment. Hemoglobin and albumin levels remaining in the brain (C) and intestine (D) after perfusion were measured to assess the extent of vasculature leakage caused by the treatments within 24 hours. Nonperfused animals treated with saline were used as a positive control. All results were expressed as mean±SEM (n=3, *P<0.05, **P<0.01, ***P<0.001, ****P<0.0001, nonsignificant [ns]).

To evaluate the potential hemorrhagic effect of our construct, bleeding time was measured after the administration of either fusion proteins or urokinase (Figure 3A). A high dose of urokinase (500 U/g) considerably prolonged bleeding time compared with saline (478±103 seconds versus 63±6 seconds, *P*<0.0001; n=3). A high dose (12 μg/g) of Mut‐scFv‐HtPlg and SCE5‐HtPlg resulted in significantly longer bleeding than the saline control (138±26 seconds and 134±32 seconds, respectively, versus 63±6 seconds; *P*<0.01 in both cases [n=3]). At 8 μg/g, the bleeding time did slightly increase but was not significantly different from saline at baseline (81±7 and 88±23 seconds, respectively, versus 63±6 seconds). At 4 μg/g, both Mut‐scFv‐HtPlg and SCE5‐HtPlg did not induce any bleeding prolongation (52±10 and 61±9 seconds, respectively, versus 63±6 seconds). We therefore selected the 4 μg/g dose for further in vivo studies. We then evaluated the systemic effect of SCE5‐HtPlg at this selected dose, 24 hours after administration, by measuring fibrinogen level in plasma (Figure 3B). Fibrinogen plasma concentration in mice treated with SCE5‐HtPlg was similar to control mice (1.34±0.12 mg/mL for the SCE5‐HtPlg group versus 1.27±0.22 mg/mL for the PBS group), whereas mice treated with urokinase had slightly reduced fibrinogen levels (0.85±0.3 mg/mL), although this reduction was not significant. We further assessed the potential effect of SCE5‐HtPlg on vasculature leakage in PBS‐perfused intestine and brain (Figure 3C and 3D). We did not observe accumulation of hemoglobin or albumin in brain samples from mice treated with both SCE5‐HtPlg and urokinase, indicating that these proteases do not harm the uninjured blood‐brain barrier. In intestine samples, a trend of albumin increase was seen in mice treated with urokinase (33.8±5.2 μg/mg total protein) compared with mice treated with saline (22.5±1.0 μg/mg total protein). Importantly, treatment with SCE5‐HtPlg had no effect on intestine vessel permeability.

### Effect on Endothelial Cell Permeability

**Figure 4:**
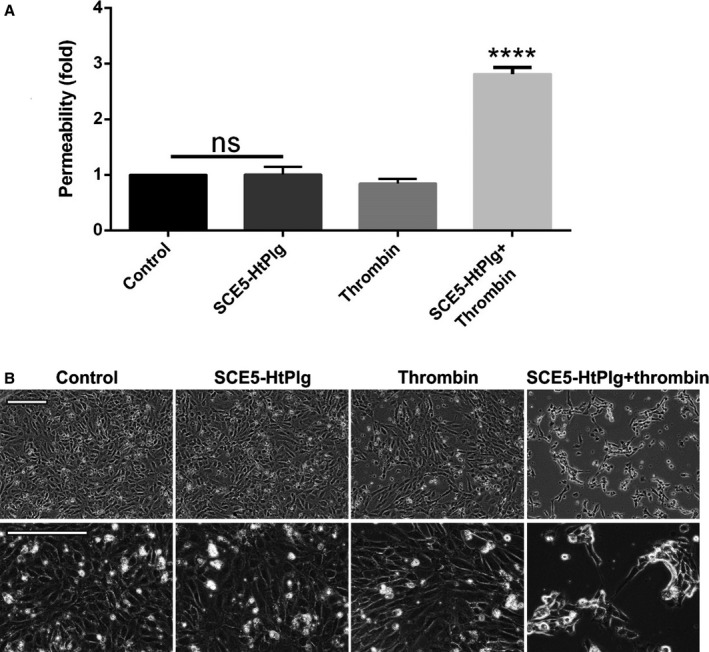
Brain microvascular endothelial cells were cultured to confluence in permeable Transwell inserts and incubated for 6 hours with anti–glycoprotein IIb/IIIa single‐chain antibody–human thrombin‐activatable microplasminogen (SCE5‐HtPlg; 100 nmol/L) only, thrombin only (2.5 U/mL), and SCE5‐HtPlg (100 nmol/L) with thrombin (2.5 U/mL). A, Permeability was measured by fluorescein isothiocyanate‐BSA passage through the monolayers over 1 hour and presented as mean±SEM values of permeability normalized to untreated controls (n=3, ****P<0.0001, nonsignificant [ns]). B, Representative phase‐contrast images of brain endothelial cells 12 hours after various treatments. Prominent gaps and morphology changes are observed in the combined treatment group, but not in cells treated with (nonactivated) SCE5‐HtPlg alone.

SCE5‐HtPlg was added alone or in combination with thrombin (to activate the construct) to confluent monolayers of brain microvascular endothelial cells and permeability compared with untreated control (used as baseline permeability) (Figure 4A). While the nonactivated construct or thrombin did not induce any permeability changes on their own (Figure 4, 1.01±0.12‐fold for SCE5‐HtPlg only and 0.84±0.07‐fold for thrombin only), addition of SCE5‐HtPlg together with thrombin induced a 2.82±0.09‐fold increase in permeability (*P*<0.0001). Microscopic examination of the cell monolayers confirmed that endothelial cells remained morphologically unaffected in the presence of the nonactivated construct (without thrombin), whereas noticeable gaps and morphological alterations were induced by the activated protease (Figure 4B).

### Targeting to the Activated GPIIb/IIIa Expressed on Human Platelets and to Ferric Chloride–Induced Thrombus

**Figure 5:**
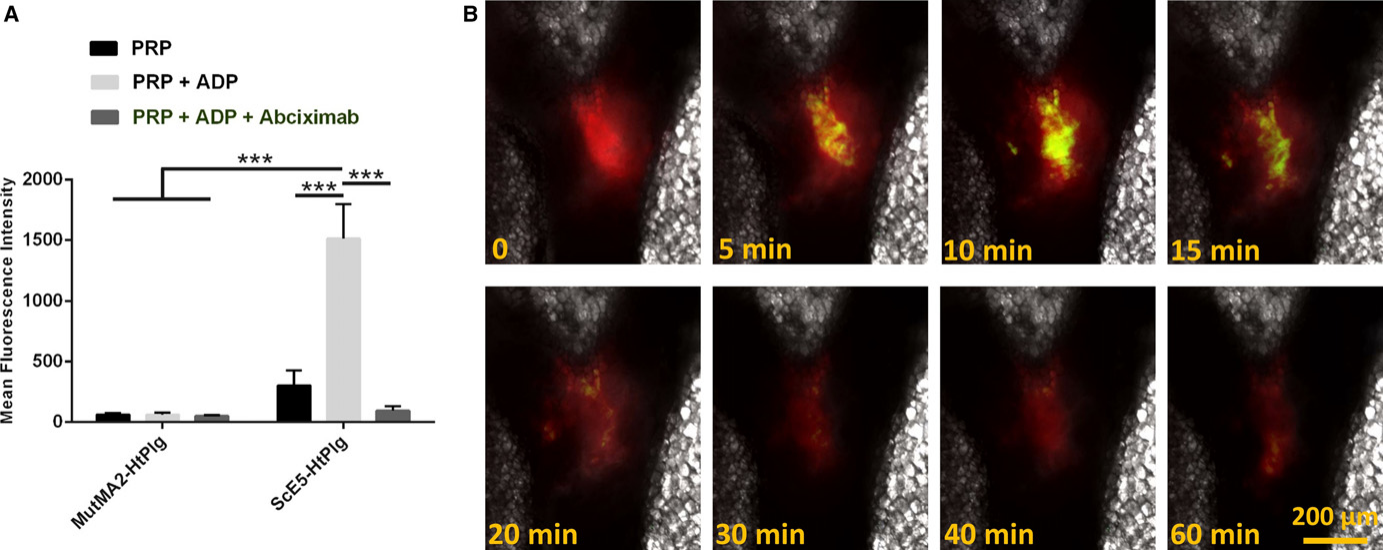
A, Flow cytometry analysis of the anti–glycoprotein (GP) IIb/IIIa single‐chain antibody–human thrombin‐activatable microplasminogen (SCE5‐HtPlg) affinity toward GPIIb/IIIa receptors on activated platelet. SCE5‐HtPlg and nontargeted control scFv HtPlg (Mut‐scFv‐HtPlg) were labeled with an anti–V5‐fluorescein isothiocyanate (FITC) antibody incubated with 3 groups of platelet‐rich plasma (PRP): nonactivated platelets (PRP), platelets activated with 20 μm ADP (PRP+ADP), and platelets activated and the GPIIb/IIIa blocked with abciximab (PRP+ADP+abciximab). The mean intensity of fluorescence associated with the platelets is shown (mean±SEM, n=5, ***P<0.001). B, The high clot specificity of the GPIIb/IIIa–targeted construct is shown by intravital microscopy on a mesentery vessel with a ferric chloride–induced thrombus after intravenous injection of SCE5‐HtPlg (4 μg/g body weight [BW]) prelabeled with an anti‐6X His tag AF488 antibody. The thrombus itself is labeled with rhodamine 6G (30 μL, 0.3% w/v). Snapshots were taken in DIC, FITC, and tetramethylrhodamine channels every 2.5 minutes from 0 to 20 minutes postinjection, then every 5 minutes for up to 1 hour postinjection. An overlay of the 3 channels is presented at representative time points.

The GPIIb/IIIa targeting ability of the SCE5‐HtPlg was assessed in vitro on human platelets and in vivo on a ferric chloride–induced thrombosis model in mouse mesentery vessels. The interaction of the SCE5‐HtPlg with resting PRP, PRP+ADP, and PRP+ADP+abciximab was assessed by flow cytometry and compared with the interaction of the nontargeted control construct Mut‐scFv‐HtPlg (Figure 5A). SCE5‐HtPlg exhibited a significantly higher mean fluorescence intensity (MFI) with activated platelets (MFI of 1514±283), as compared with nonactivated platelets (MFI of 302±126) or activated then blocked platelets (MFI of 94±38) (*P*<0.001, n=5). The Mut‐scFv‐HtPlg construct did not show any increase in fluorescent signal uptake when incubated with the same 3 PRP groups (MFI of 62±12 with PRP, 60±18 with PRP+ADP, and 50±8 with PRP+ADP+abciximab).

The SCE5‐HtPlg was then labeled with an anti–His‐AF488 antibody and injected intravenously into a mouse subjected to a ferric chloride–induced thrombus on the mesentery vessel. Figure 5B shows intravital fluorescent microscopy observations of the thrombus observed in the tetramethylrhodamine channel (shown in red) before (t=0) and after (t=5, 10, 15, 20, 30, 40, 60 minutes) the injection of the AF488‐labeled SCE5‐HtPlg construct observed in the FITC channel (shown in green). An accumulation of SCE5‐HtPlg was observed over 15 minutes postinjection at the site of the thrombus, which indicates efficient clot targeting properties in vivo.

### In Vivo Thrombolysis Study in 2 Thrombosis Models

**Figure 6:**
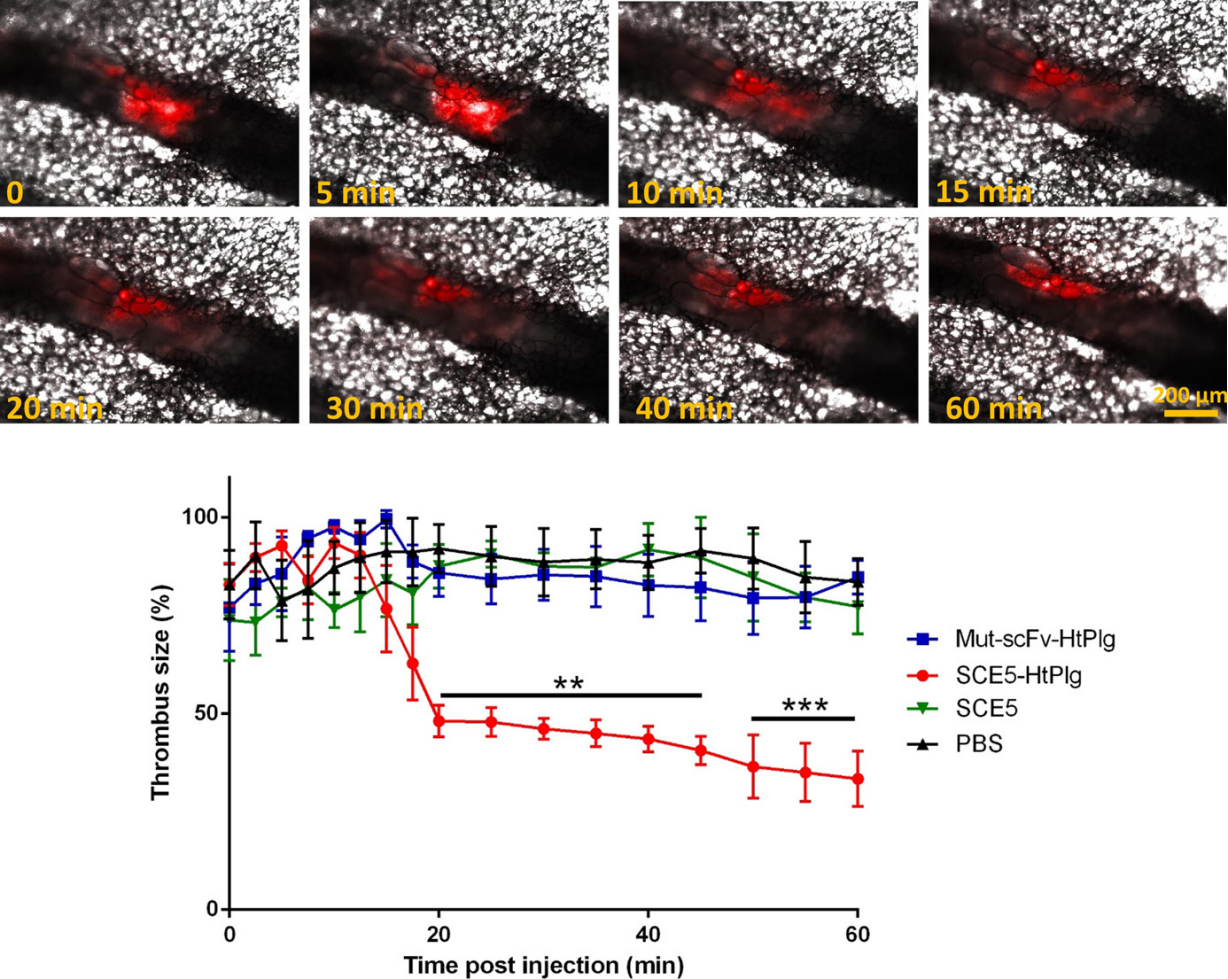
Thrombolysis is shown by intravital microscopy on mesenteric vessels with a ferric chloride–induced thrombus after intravenous injection of anti–glycoprotein IIb/IIIa single‐chain antibody (SCE5)–human thrombin‐activatable microplasminogen (SCE5‐HtPlg; 4 μg/g body weight [BW]). The thrombus is labeled with rhodamine 6G (30 μL, 0.3% w/v). Snapshots were taken in differential interference contrast and tetramethylrhodamine (TRITC) channels every 2.5 minutes from 0 to 20 minutes postinjection then every 5 minutes for up to 1 hour postinjection. An overlay of the 2 channels at representative time points is presented. The size of the thrombus was measured at each time point on the TRITC channel (yellow dotted lines) and the percentage of thrombus degradation obtained with nontargeted control scFv HtPlg (Mut‐scFv‐HtPlg; 4 μg/g BW), SCE5‐HtPlg (4 μg/g BW), SCE5 only (1.7 μg/g BW), or PBS treatment was plotted over the time postinjection (mean±SEM, n=3, **P<0.01, ***P<0.001). Scale bar 200 μm.

The thrombolytic capacities of this new drug were tested on a ferric chloride thrombosis mouse model induced on an exteriorized mesentery. Platelets and leukocytes were labeled with rhodamine 6G, which enabled observation of the thrombus by fluorescent intravital microscopy. Thrombolytic treatment was injected intravenously when the thrombus caused more than 50% occlusion. The size of the thrombus was monitored over time after injection of the targeted fusion protein and the effect was compared with the nontargeted control at the same dose, with the SCE5 only at equimolar dose, and with saline. Figure 6 shows a thrombus identified in the tetramethylrhodamine fluorescent channel (in red color) before (t=0) and after (t=5, 10, 15, 20, 30, 40, 60 minutes) the injection of SCE5‐HtPlg at 4 μg/g. The relative size of the clot reduced progressively from 10 minutes after the injection and became significantly different from saline control at 20 minutes (48±3% versus 92±5%, *P*<0.01, n=3), then slowly reached 36±7% at 50 minutes after treatment (different from 90±6% with saline *P*<0.001). Injection of Mut‐scFv‐HtPlg at the same dose or SCE5 only at equimolar dose did not induce any degradation; the thrombus reached stable occlusion after injection, similar to saline treatment (Figure S3).

**Figure 7:**
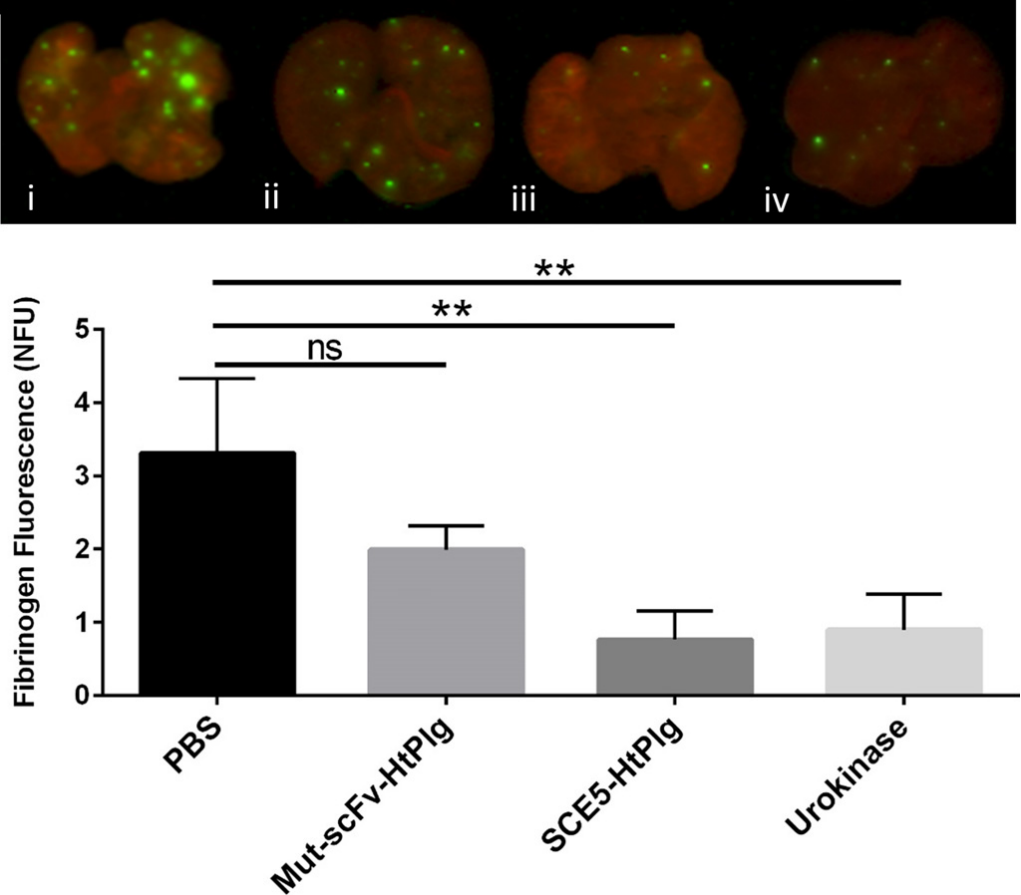
In vivo fibrinolysis study in a mouse model of pulmonary embolism. Emboli were induced and fluorescently stained by infusing a mixture of Innovin (recombinant tissue factor and synthetic phospholipids) and Cy‐7–labeled fibrinogen. Four groups of drugs were injected 10 minutes after: PBS, nontargeted control scFv HtPlg (Mut‐scFv‐HtPlg; 4 μg/g body weight [BW]), anti–glycoprotein IIb/IIIa single‐chain antibody (SCE5)–human thrombin‐activatable microplasminogen (SCE5‐HtPlg; 4 μg/g BW), and urokinase (500 U/g BW). Mice were sacrificed 50 minutes after drug administration, perfused with PBS, and lungs were harvested and scanned using an Odyssey Infrared Imaging System (700 nm channel shown in red, 800 nm channel shown in green). Fibrinogen fluorescence is measured and presented as a mean value of normalized fluorescence units (mean±SEM, n=3, not significant [ns], **P<0.01).

The efficacy of the fusion protein to lyse thrombi in vivo was then confirmed in a mouse model of pulmonary embolism (Figure 7). Ten minutes after the induction of thrombosis in the lung of mice via intravenous injection of Innovin mixed with Cy7‐labeled human fibrinogen, 4 different treatments were tested: PBS, Mut‐scFv‐HtPlg, SCE5‐HtPlg, and urokinase. The injection of nontargeted thrombin‐cleavable plasminogen also decreased the amount of fibrin in the lung; however, it did not show a significant reduction compared with PBS treatment (fluorescent ratio of 1.99±0.32 versus 3.43±1.09, *P*=0.18). The SCE5‐HtPlg treatment resulted in a 4‐fold reduction of fibrinogen fluorescence in the lung (0.76±0.39 versus 3.43±1.09, *P*<0.01). This value was similarly efficient as urokinase treatment (0.89±0.49 versus 3.43±1.09, *P*<0.01).

## Discussion

In this study, we developed a new fibrinolytic fusion protein activated by thrombin into microplasmin and specific to activated GPIIb/IIIa receptors expressed on activated platelets. The targeted (SCE5‐HtPlg) and nontargeted (Mut‐scFv‐HtPlg) constructs exhibited similar sizes between 75 and 55 kDa on gel electrophoresis analysis. We attribute the higher size to glycosylation, as often observed from protein production in mammalian cells.^32^ Both proteins were effectively cleaved in vitro following incubation with thrombin. Results from anti‐V5 Western blot analyses revealed progressive degradation of the full constructs, releasing a smaller fragment that corresponded to microplasmin.^33^ The amidolytic assay confirmed that the microplasmin thereby released was able to cleave a plasmin substrate, whereas no plasmin activity was detected in the absence of thrombin‐induced cleavage. The thrombin‐specific plasmin activity of the SCE5‐HtPlg and the Mut‐scFv‐HtPlg was therefore demonstrated. Importantly, the SCE5‐HtPlg was not activated by tPA, urokinase, or TAFIa, further highlighting the specificity of this construct to thrombin.

The in vitro thrombolytic study revealed that the fusion proteins are effective to lyse thrombi obtained from coagulation of human blood. There was no statistical significant difference between SCE5‐HtPlg and Mut‐scFv‐HtPlg in the thrombolytic capacity when tested at the same dose. This implies that the SCE5 portion itself does not contribute to the lysis effect observed in static conditions. However, the maximum degradation obtained with the HtPlgs is on average limited to 90% at 0.2 mg/mL and at 40% with 0.1 mg/mL, whereas the addition of urokinase led to full degradation at all concentrations tested. We believe these different lysis profiles reflect the different pathway affected by our fibrinolytic molecule. Urokinase converts the endogenous substrate (plasminogen) into plasmin, while HtPlg acts directly as microplasmin activated by thrombin generated locally. Thus, the concentration of urokinase may impact the rate of plasmin generation but not the final effective concentration, whereas the concentration of the HtPlg will effectively be limited by the amount of microplasmin. We attributed the plateau observed with HtPlg and plasmin treatments to the presence of plasmin inhibitors in the blood (α_2_‐macroglobulin and α_2_‐antiplasmin).^34^, ^35^ The fibrinolysis profile obtained in vitro would therefore reflect better control over the plasmin generated locally and over its neutralization.

Tail bleeding experiments have shown that the systemic administration of SCE5‐HtPlg and Mut‐scFv‐HtPlg induces bleeding prolongation in a dose‐dependent manner. Urokinase at the therapeutic dose established in a previous study (500 U/g BW^23^) resulted in a highly significant increase in bleeding time, even higher than the prolongation obtained with 12 μg/g BW doses of the HtPlg constructs. This observation supports the theory that targeting the activated GPIIbIIIa receptor only provides a better localization of plasmin generation compared with systemic fibrinolysis and consequently results in lower hemostatic plug disruption at sites of vascular injury. In addition, the same hemostatic safety advantage over plasminogen activator has been reported with the use of direct fibrinolytic (mainly plasmin and microplasmin).^36^, ^37^ At the 4 μg/g BW dose, SCE5‐HtPlg did not consume plasma fibrinogen and was not associated with any brain hemorrhage or gastrointestinal effect at 24 hours after administration in healthy animals. The fear of hemorrhagic complications is the main obstacle for the use of plasminogen activators in clinical settings.^38^ This risk is even more prominent as a large portion of patients admitted for thrombolytic therapy have received antiplatelet therapy.^39^ The safety profile presented for the SCE5‐HtPlg is therefore highly favorable for clinical translation. However, it should be noted that the present study is limited to the evaluation of bleeding risk in healthy animals, whereas hemorrhagic complications seem to predominantly occur in ischemic or thromboembolic conditions.^40^, ^41^

These findings are also comparable with the dose‐escalation clinical trial outcomes of the thrombin‐cleavable plasminogen mutant developed by Vernalis Biotech (V10153). The VASTT (V10153 Acute Stroke Thrombolysis Trial) has been halted because 3 of 9 patients in the 7.5‐mg/kg group developed significant hemorrhagic complications.^16^ The TIMI 31 (Thrombolysis in Myocardial Infarction Trial) had a better outcome, with 34% of patients treated with 5, 7.5, and 10 mg/kg achieving complete flow in the infarct‐related artery.^13^ However, the margin between efficacy and bleeding still appears tight since, at these same doses, 7% of the patients sustained TIMI major bleeding events and 14% sustained TIMI minor or minimal bleeds. In fact, our in vitro fibrinolysis study in static conditions suggested the same limit in terms of risk‐benefit ratio as the efficient dose of 0.2 mg/mL determined in vitro would correspond to a 12 μg/g BW dose in vivo (approximating the blood volume as 6% of the body weight), which has shown bleeding prolongation. Hence, even though the thrombin activation feature may effectively reduce the risk of hemorrhage over plasminogen activators, it was necessary to enhance the clot specificity of the HtPlg by recombinant fusion to a single‐chain antibody and lower the dose required.

We demonstrated by flow cytometry that the SCE5‐HtPlg construct has a strong affinity for human activated platelets, specific to surface‐bound activated GPIIb/IIIa receptors since the fluorescence uptake was completely blocked when platelets were preincubated with a GPIIb/IIIa blocker (abciximab). The targeting behavior was also verified in vivo on a ferric chloride–induced thrombosis model on mouse mesenteric vessel. The FITC signal detected at the site of the thrombus observed after the injection of 4 μg/g BW of SCE5‐HtPlg labeled with an anti‐His tag AF488 antibody suggests a clot‐specific accumulation of the construct. Upon activation, the microplasmin portion, which contains the 5x histidine repeat at the C‐terminus is cleaved from the SCE5 portion. Thus, this experiment indicates that most of the SCE5‐HtPlg was cleaved into microplasmin within 20 minutes postinjection, as the FITC signal decreased from the 20‐minute time point.

A strong in vivo fibrinolytic effect was observed on the same ferric chloride thrombosis model in mice treated with the SCE5‐HtPlg at a dose of 4 μg/g BW, whereas no degradation was observed with the nontargeted control, which confirms the necessity of the targeting behavior to obtain efficient thrombolysis at this low dose. Similarly, the SCE5 itself, at equimolar dose, did not result in any degradation. In the lung embolism model, we compared the SCE5‐HtPlg with a treatment of urokinase that is currently used in the clinic for fibrinolytic therapy for lung embolism^42^ and a similar 4‐fold reduction of thrombosis was measured versus the saline control treatment. Interestingly, although no significant difference was measured, the same dose of nontargeted construct seemed to slightly reduce the amount of emboli in this model. We attribute the variations in fibrinolysis effect to a presumable different nature of thrombi between the two models. Although the mechanisms underlying ferric chloride–induced thrombosis are not completely elucidated, it is reported to result in the formation of platelet‐rich thrombi resistant to lysis.^43^, ^44^ Therefore, in a ferric chloride–induced model, the thrombi were resistant to lysis from the Mut‐scFv‐HtPlg but with the SCE5‐HtPlg injected at the same dose, the platelet targeting property enabled good accumulation of the drug at the site of the clot and thereby potentiated the degradation. On the other hand, in the lung embolism model, the thrombosis is induced by tissue factor, which triggers the clotting cascade via the extrinsic pathway and has been shown to form fibrin‐rich clots.^45^ The thrombolysis activity of HtPlg and urokinase being based on degradation of fibrin, these 3 treatments were accordingly more potent in this model but the platelet‐targeting properties of the SCE5‐HtPlg enhance the activity to a lower extent. Our in vivo experiments indicate that SCE5‐HtPlg is an effective thrombolytic drug capable of lysing platelet‐rich as well as fibrin‐rich thrombi, which corresponds to the thrombus composition encountered in patients with non–ST‐segment elevation myocardial infarction and ST‐segment elevation myocardial infarction for which thrombolytic therapy is currently recommended.^46^, ^47^, ^48^

Other issues reported with plasminogen activators are the detrimental impact on the neurovascular unit and alteration of the blood‐brain barrier.^30^, ^49^, ^50^ Our in vitro permeability assay revealed that the SCE5‐HtPlg affects primary human brain microvascular endothelial cells, but only when activated with thrombin. This result suggests that no undesired side effect would be observed on the endothelium away from thrombosis sites. We also consider these results encouraging because at a similar dose range (25–250 nmol/L), recombinant tPA added with no substrate was found to induce substantial permeability increases in previous studies.^30^

This new thrombolytic agent also presents the advantage to, presumably, be unaffected by circulating TAFI and PAI‐1, which have been identified as major causal factors of fibrinolysis failure.^51^, ^52^, ^53^, ^54^ Indeed, both of these fibrinolysis inhibitors inhibit or indirectly reduce the action of plasminogen activators, which we bypassed with our approach. We verified that the same concentration of PAI‐1 and TAFIa significantly delayed in vitro thrombolysis from urokinase but not from SCE5‐HtPlg.

One concern regarding the strategy of thrombin activation was the quantity of thrombin available at the thrombosis site. In this study, the efficient lysis with the SCE5‐HtPlg indicates that the thrombin generated in 2 different thrombosis models in mice is sufficient to ensure adequate activation. However, the settings of these animal experiments are very specific. In both studies, we injected the drug as a single bolus within a relatively short time after the induction of thrombosis. Therefore, further investigations are required to evaluate the efficacy of the SCE5‐HtPlg as an acute thrombosis treatment at several time points after thrombus induction. Although few studies describe the thrombin activity within a thrombus after onset, recent research shows an accumulation of thrombin within the core of the clot, bound to fibrin fibers, protected from thrombin inhibition and possibly contributing to the prothrombotic nature of thrombi.^55^, ^56^ Clinical trial outcomes with the thrombin inhibitor abigatran in stroke treatment up to 12 hours after the onset also suggest that thrombin remains an important player at later stages of acute thrombosis.^57^, ^58^ Therefore, our new fibrinolytic drug could be effective several hours after thrombosis formation.

## Conclusions

This newly proposed thrombolytic drug provided in vivo thrombolysis equivalent to a standard fibrinolytic drug commonly used in the clinic, exhibited a better safety profile in regards to hemorrhagic complications, and has the potential to overcome the main limitations of thrombolytic therapy.

## Sources of Funding

This work was funded by the National Health and Medical Research Council (NHMRC). Bonnard has received funding from the People Programme (Marie Curie Actions) of the European Union's Seventh Framework Programme (FP7/2007‐2013) under REA grant agreement No. 608765, Niego is supported by a postdoctoral fellowship from the National Heart Foundation of Australia (award No. 100906). Alt was supported by the German Research Foundation (Al 1521/1‐1), Peter is a Principal Research Fellow of the NHMRC, and Hagemeyer is a National Heart Foundation Career Development Fellow. The work was also supported in part by the Victorian Government's Operational Infrastructure Support Program and Victoria's Science Agenda Strategic Project Fund.

## Disclosures

Peter is an inventor on patents describing activated platelet–targeting recombinant antibodies. All other authors have declared that they have no conflicts of interest to disclose.

## Supporting information


**Figure S1.** A, Vector map of the nontargeted human thrombin cleavable microplasminogen plasmid (Mut‐scFv‐HtPlg) and the human thrombin cleavable microplasminogen targeted toward activated GPIIb/IIIa (SCE5‐HtPlg). Electrophoresis with 0.8% agarose gel. DNA fragments were digested using restriction enzymes NotI and Xho. Mut‐scFv‐HtPlg (≈1.8 kbp), SCE5‐HtPlg (≈1.8 kbp) and HtPlg (≈0.8 kbp) only after polymerase chain reaction amplification. The undigested (uncut) pSectag vector (negative control) containing the SCE5 single‐chain antibody runs at 1 kbp. B, 12% SDS‐PAGE and Western blot analysis using a horseradish peroxidase coupled to anti‐6X His‐tag antibody of the Mut‐scFv‐HtPlg and SCE5‐HtPlg.
**Figure S2.** The effect of plasminogen activator inhibitor‐1 (PAI‐1) and thrombin activatable fibrinolysis inhibitor (TAFIa) on the fibrinolytic capacities of urokinase and of SCE5‐HtPlg were tested in vitro on thrombi formed in halo shape at the bottom of 96‐well plates. Urokinase at 200 U/mL (A) and SCE5‐HtPlg at 0.2 mg/mL (B) was tested in several conditions: (1) incubated 20 minutes in saline and added to blood clots obtained from whole blood, (2) incubated 20 minutes with 6 nmol/L of PAI‐1 and added to blood clots obtained from whole blood, (3) incubated 20 minutes in saline and added to blood clots obtained from whole blood supplemented with TAFIa (20 nmol/L). The degradation of the thrombi was monitored over 1 hour at 37°C by spectrophotometry from the absorbance of the blood covering progressively the center of the well. For each assay, the positive control for the assay contains blood topped up to the final volume with buffer while the negative control contains a pre‐prepared halo aggregate topped up with buffer to the final volume. Mean thrombus degradation±SEM is plotted over time (n=4). C, Mean thrombus degradation initiation time is presented as mean value±SEM (n=4, *P<0.05, ns: nonsignificant).
**Figure S3.** Intravital microscopy observations on a vein with a ferric chloride–induced thrombus after intravenous injection of SCE5‐HtPlg (4 μg/g body weight [BW]) (A), MutMA2‐HtPlg (4 μg/g BW) (B), SCE5 (1.7 μg/g BW) (C) or PBS (D). The thrombus is labeled with rhodamine B. Snapshots were taken in DIC and tetramethylrhodamine (TRITC) channel every 2.5 minutes from 0 to 20 minutes postinjection then every 5 minutes up to 1 hour postinjection. An overlay of the 2 channels at representative time points are presented. Scale bar 200 μm.
