# Supplementary material for: Novel Thrombolytic Drug Based on Thrombin Cleavable Microplasminogen Coupled to a Single‐Chain Antibody Specific for Activated GPIIb/IIIa
Source: J Am Heart Assoc. 2017 Feb 3;6(2):e004535. doi: 10.1161/JAHA.116.004535 (PMC5523756; doi:10.1161/JAHA.116.004535)
Supplement: Supplementary file 1 — Figure S1. A, Vector map of the nontargeted human thrombin cleavable microplasminogen plasmid (Mut‐scFv‐HtPlg) and the human thrombin cleavable microplasminogen targeted toward activated GPIIb/IIIa (SCE5‐HtPlg). Electrophoresis with 0.8% agarose gel. DNA fragments were digested using restriction enzymes NotI and Xho. Mut‐scFv‐HtPlg (≈1.8 kbp), SCE5‐HtPlg (≈1.8 kbp) and HtPlg (≈0.8 kbp) only after polymerase chain reaction amplification. The undigested (uncut) pSectag vector (negative control) containing the SCE5 single‐chain antibody runs at 1 kbp. B, 12% SDS‐PAGE and Western blot analysis using a horseradish peroxidase coupled to anti‐6X His‐tag antibody of the Mut‐scFv‐HtPlg and SCE5‐HtPlg. Figure S2. The effect of plasminogen activator inhibitor‐1 (PAI‐1) and thrombin activatable fibrinolysis inhibitor (TAFIa) on the fibrinolytic capacities of urokinase and of SCE5‐HtPlg were tested in vitro on thrombi formed in halo shape at the bottom of 96‐well plates. Urokinase at 200 U/mL (A) and SCE5‐HtPlg at 0.2 mg/mL (B) was tested in several conditions: (1) incubated 20 minutes in saline and added to blood clots obtained from whole blood, (2) incubated 20 minutes with 6 nmol/L of PAI‐1 and added to blood clots obtained from whole blood, (3) incubated 20 minutes in saline and added to blood clots obtained from whole blood supplemented with TAFIa (20 nmol/L). The degradation of the thrombi was monitored over 1 hour at 37°C by spectrophotometry from the absorbance of the blood covering progressively the center of the well. For each assay, the positive control for the assay contains blood topped up to the final volume with buffer while the negative control contains a pre‐prepared halo aggregate topped up with buffer to the final volume. Mean thrombus degradation±SEM is plotted over time (n=4). C, Mean thrombus degradation initiation time is presented as mean value±SEM (n=4, *P<0.05, ns: nonsignificant). Figure S3. Intravital microscopy observations on a vein with a ferric ch [file JAH3-6-e004535-s001.pdf]

SUPPLEMENTAL MATERIAL

Generation, expression and purification of single-chain antibodies fused with human thrombin activatable plasminogen

The DNA sequence coding for the human thrombin activatable microplasminogen (HtPlg) was designed from the sequence of human microplasminogen[1] in which the sequence CCT GGA AGG GTT GTA GGG GGG (nucleotides 49 to 69) has been replaced by this sequence ACC ACC AAA ATT AAA CCG CGT ATT GTT GGT GGT, and obtained from GeneArt™ (ThermoFisher Scientific, US). The HtPlg construct was then fused with two different single-chain antibodies, the activated GPIIb/IIIa-targeted (SCE5) and non-targeted (Mut-scFv) previously described[2], subcloned into the pSecTag vector system. After amplification by polymerase chain reaction (PCR), DNA fragments were digested using restriction enzymes *NotI* and *XhoI* (NEB, US) then ligated together using T4 ligase (New England BioLabs, UK) at 16°C overnight. The resulting plasmid constructs were then transformed into BL21 Star *E.coli* cells (Invitrogen, US). The DNA amplified by PCR and restriction digests was analysed by electrophoresis on a 0.8% agarose gel.

The fusion constructs SCE5-HtPlg and Mut-scFv-HtPlg were transfected with polyethylenimine (PEI, Polyscience Inc., Germany) for expression in human embryonic kidney cells (freeStyleHEK 293-Fcells, Life Technologies, US) suspension cells grown in a CO_2 incubator at 37ºC, shaking at 110 rpm. DNA plasmid for transfection was diluted with PEI and added to culture suspensions following the ratio of 1µg DNA:3µg PEI:1mL culture. H293F cells were adjusted at $2x10^6$ cells/mL with Freestyle 293 expression medium (Invitrogen, US) with a viability greater than 95 %. The culture was supplemented with 5g/L Lupin after 1 and 5 days. At day 3, 5 and 7

after transfection, the culture was supplemented with 2mM glutamine. The glucose level was maintained at a final concentration of 6g/L. The cells were harvested when viability was 50 %. The suspensions were centrifuged at 14,000g for 15 minutes and the supernatants were collected for protein purification.

Both SCE5-HtPlg and Mut-scFv-HtPlg proteins carry a 6x His-tag at the C-terminal end of their amino acid sequence for purification and a V5-tag from their single-chain antibody part (SCE5 and Mut-scFv). Proteins were purified by fast liquid protein chromatography with a nickel-based metal affinity column Ni-NTA (Invitrogen, US). Protein concentration was determined with Direct Detect Infrared Spectrometer (Merck Millipore, US). Purity of the proteins was analysed by SDS-PAGE gel stained with Coomassie Brilliant Blue visualized with Odyssey imaging system (LI-COR Biosciences, US) in the 700 channel. Western blot analysis was performed to confirm the presence of the SCE5-HtPlg and Mut-scFv-HtPlg fusion proteins by revealing the 6xHis-tag and the V5-tag. After SDS-gel electrophoresis, the proteins were transferred on PVDF membranes which were blocked with 5% skimmed milk at 4°C overnight then incubated 1 hour with Anti-6xHis-tag antibody HRP (horse radish peroxidase) or anti-V5-tag antibody HRP. Secondary hybridization was performed with SuperSignal West Pico chemiluminescent (ECL) substrate (Thermo Scientific Inc, US) for the HRP enzyme. ECL signal on membranes were visualized using a BioRad Gel-Doc system.

Cleavage of the thrombin activatable microplasminogen proteins into microplasmin

The cleavage of SCE5-HtPlg and Mut-scFv-HtPlg from thrombin incubation into microplasmin was studied *in vitro* with western blot analysis and spectrophotometry.

Both proteins were incubated at 200 µg/mL with 3U/mL of Thrombin (Siemens, Germany) in 150 mM Tris-HCl buffer (pH=8) at 37°C. At t=0, 10, 20, 30, 40, 50 and 60 minutes incubation, 10 µL samples were aliquoted, mixed with 30 µL PBS and 10 µL 5x sample buffer with DTT, heated at 95 °C then analysed with Western blot analysis with V5-tag revelation.

Generation of microplasmin was monitored from incubation with thrombin by spectrophotometry with the S2251 amidolytic assay. ScE-HtPlg and Mut-scFv-HtPlg proteins were plated in a 96 well plate at 13 µg/mL in 150 mM Tris-Hcl buffer (pH=8) with different thrombin concentrations (0, 0.2, 1 and 2 Units/mL) and S2251 plasmin substrate (Chromogenix, US) at 0.3 mM. The microplasmin generation was measured from the increasing absorbance induced by the cleavage of the S2251 substrate cleavage at 410 nm with a Plate reader (EnSpire Multimode, PerkinElmer, US). Measurements were started just after thrombin addition and taken every minute over 3 hours at 37°C, with shaking in between each time point. Positive control was assessed with human plasmin (Sigma-Aldrich, US) at different concentrations (0, 0.004, 0.008, 0.012, 0.016, 0.02 U/mL) incubated with 0.3 mM S2251 substrate. Microplasmin generation was also monitored after the addition of 2 U/mL urokinase, 2 nM recombinant human tissue plasminogen activator (tPA, Boehringer Ingelheim GmbH, Germany) and 16 µg/mL activated thrombin activatable fibrinolysis inhibitor (TAFIa, Sigma, US). Negative control was assessed with thrombin only at different concentrations (0, 0.2, 1 and 2 Units/mL), urokinase (2U/mL) only, tPA only (2 nM) and TAFIa only (16 µg/mL) incubated with 0.3 mM S2251 substrate.

96 well plate fibrinolysis assay

Blood from 8 healthy volunteers was collected in sodium citrate 3.8 % (w/v). Thrombi were formed in halo shape at the bottom of 96 well plates with 25 µL of blood mixed with 3.75 µL Innovin (Dade® Innovin®, Tissue factor with phospholipids, Siemens) diluted 5 times from reconstitution prepared according to manufacturer's instruction and 1.25 µL of 0.25 M Calcium chloride. The degradation of the halo thrombi was measured with a plate reader (EnSpire Multimode, PerkinElmer, US) from the absorbance of the blood covering progressively the center of the well. The fibrinolysis rate was assessed by one measurement at 510 nm every minute with shaking in between each time point over 1 hour at 37°C, starting just after the addition of thrombolytic drugs contained in 70 µL. Different concentration of Plasmin (0.01 U/mL, 0.1 U/mL and 0.5 U/mL), urokinase (100, 200, 400 U/mL), SCE5-HtPlg (0.1 and 0.2 mg/mL) or Mut-scFv-HtPlg (0.1 and 0.2 mg/mL) were tested (n=4). Additional experiments were performed with urokinase at 200 U/mL and SCE5-HtPlg at 0.2 mg/mL preincubated 20 minutes at room temperature with 6 nM of human plasminogen activator inhibitor-1 (PAI-1, Molecular Innovation, US) and on thrombi obtained from blood supplemented with 20 nM of activated thrombin activatable fibrinolysis inhibitor (TAFIa, Sigma, US). Initiation times were measured on the degradation profiles for each run. Negative controls were obtained by addition of 70 µL of PBS with no thrombolytic. Positive controls were obtained from well prepared with 25 µL of blood fluid topped up with 75 µL of PBS. The positive control wells provided absorbance values corresponding to full degradation (A_{total}) and the negative control wells provided reading for no degradation (A_{zero}). At each time point, the percentage of degradation were obtained from this formula: D(t)=100*(A(t) –

$A_{zero}(t))/(A_{total}(t)-A_{zero}(t))$. Replicates were obtained with thrombi made from the blood of 4 different donors. Mean percentage of degradations ± SEM are plotted over time.

Flow cytometry

Blood from 5 healthy adult volunteers was collected in sodium citrate 3.8 % (w/v). Platelets-rich plasma (PRP) was obtained by centrifugation at 180g for 10 min and diluted 1 in 10 in PBS containing Ca^{2+} and Mg^{2+} ions. Activated PRP was obtained by stimulation of PRP with 20 µM of ADP (adenosine diphosphate). Activated then GP IIb/IIIa blocked platelets were obtained by incubation with abciximab at high concentration (50 µg/mL, ReoPro). Before assessing the interaction with the fusion proteins, tubes of PRP, PRP+ADP and PRP+ADP+Abciximab were prepared and incubated with PAC-1 FITC antibody (BD Biosciences) to assess GP IIb/IIIa expression on the platelet surface. We thus confirmed for each test that the GP IIb/IIIa complex is detected on platelets from the PRP+ADP group but not on platelets from the PRP or PRP+ADP+Abciximab groups. The tubes of interest were prepared with 50 µL of diluted PRP, PRP+ADP or PRP+ADP+Abciximab incubated for 20 minutes with 1 µL of SCE5-HtPlg and Mut-scFv-HtPlg (20 µg/mL), together with 1 µL of anti-V5-FITC antibody (0.1 mg/mL, Invitrogen, US) to label the protein. Samples were fixed with 1x Cellfix (BD Bioscience, US) and analysed on a FACSCanto™ II Flow cytometer (BD Biosciences, US) with 10,000 events collected per samples. For each experiment, platelet populations were gated according to their typical granulometry measured on forward scattered light/size scattered light plots. The FITC Mean fluorescent intensity (MFI) measured within these identified platelet populations thus correspond to the interaction between FITC labelled SCE5-HtPlg or Mut-scFv-HtPlg and platelets. Results were presented as mean values of MFI ± SEM (n=5).

Template tail bleeding

All experiments involving animals were approved by the Alfred Medical Research and Education Precinct Animal Ethics Committee (E/1534/2015/B and E/1589/2015/B). Six weeks old male C57BL/6 mice were anesthetized with ketamine (50 mg/kg; Parnell Laboratories, Australia) and xylazine (10 mg/kg, Troy Laboratories, Australia) and placed on a 37 °C heater mat to prevent hypothermia. Mouse bleeding time was measured by tail template method. Several group of drug were injected intravenously; Urokinase at 100 and 500 Unit per gram body weight (/g BW), SCE5-HtPlg at 2, 4, 8 µg/g BW, Mut-scFv-HtPlg at 2, 4, 8 µg/g BW and saline (n=3). 30 seconds after the drug administration, a longitudinal incision, 2 mm deep, 4 mm long, was made, starting 10 mm from the beginning of the tail. Care was taken to ensure incision was made over the superficial tail vein running along the left axis of the tail. Bleeding time was recorded between the section and the arrest of bleeding. Results was expressed as mean values ± SEM (n=3).

Hemoglobin, albumin and plasma fibrinogen measurements

Six weeks old male C57BL/6 mice were injected *IV* with Urokinase at 500 U/g BW, SCE5-HtPlg at 4 µg/g BW and saline (n=3). 24h after drug administration, mice were anesthetized and 600 µL of blood was collected in 3.2% citrate and centrifuged 15 min at 2,000g to isolate plasma. The concentration of fibrinogen in plasma was determined with a mouse fibrinogen antigen ELISA kit (Molecular Innovations, US). Mice were then gently perfused with 30 mL of saline then brain and intestine were harvested. Similar parts of each tissue were isolated, weighted and lysed in Triton X-100 solution (1% v/v in PBS). An additional group of 3 mice treated with saline but not perfused were used as a positive control. Hemoglobin and albumin levels were

measured in brain and intestine lysates by spectrophotometry using a hemoglobin substrate (Quantichrom Hemoglobin, Bioassay Systems, US) and by a mouse Albumin ELISA test (Bethyl Laboratories, US) respectively, and were expressed per gram of protein in the lysate measured by bicinchoninic acid assay.

Cell permeability assay

This experiment was adapted from a previously described cell permeability assay which mimics *in vitro* blood-brain barrier function[3]. Primary human brain microvascular endothelial cells (hBEC; line ACBRI 376, Cell-System Corporation) were seeded in collagen-I-coated Transwell inserts (6.5mm, polyester membrane with 0.4um pores; Corning) at 20,000 cells per insert. Cells were grown to confluence over 3 days in MV2 endothelial cell medium (PromoCell) with 50ug/ml gentamicin. Following one wash in serum-free medium, cells were stimulated in the luminal compartment with SCE5-HtPlg alone (100nM), bovine thrombin alone (2.5 U/ml; plasminogen-free; Merck) or their combination. 6 h post stimulation, permeability changes were assessed by measurement of fluorescein isothiocyanate-conjugated bovine serum albumin passage from the luminal to the abliminal compartment, as previously described[3, 4]. Results were expressed as fold induction from untreated inserts.

Ferric chloride induced thrombosis on mesenteric vessel

Targeting and thrombolytic capacities of the thrombin activatable microplasminogen fusion proteins were tested on a mouse model of thrombosis induced by Ferric chloride superfusion on mesenteric vessel performed as described previously[5].

Briefly, six weeks old male C57BL/6 mice were anesthetized with ketamine (50 mg/kg) and xylazine (10 mg/kg) and placed on a 37 °C heater mat to prevent hypothermia. The mesentery was exteriorised through a midline abdominal incision. Rhodamine 6G (30 µL, 0.3% w/v, Sigma) was injected IV to label leukocytes and platelets. A filter paper (1mm x 2mm) saturated with 6% ferric chloride was placed on an isolated mesenteric vessel for 2 minutes to induce vessel wall injury and subsequent thrombus formation. Real time formation of the thrombosis was monitored by intravital microscopy on an Olympus IX81 inverted microscope in the TRITC fluorescent channel to visualise the thrombus stained with Rhodamine 6G and in the Differential Interference Contrast (DIC) channel to identify the vessel wall. When the thrombus reached over 50 % occlusion, 4 groups of drug were injected intravenously; SCE5-HtPlg at 4 µg/g BW, Mut-scFv-HtPlg at 4 µg/g BW, SCE5 at 1.7 µg/g BW (equimolar dose) and saline (n=3). Snapshots were taken in DIC and TRITC channel every 2.5 minutes from 0 to 20 min post injection then every five minutes up to 1 hour post injection. To avoid any photo bleaching of the fluorescently stained thrombus, exposure to light was fully prevented in between each snapshot. Thrombus size was measured at each time point (Thr_t) from TRITC channel images converted to binary images with ImageJ software (NIH, US). For each experiment, the biggest size measured for the thrombus was identify (Thr_{max}). The relative thrombus size was obtained from the formula $100*Thr_t/Thr_{max}$ and the mean values ± SEM are plotted over the time post-injection (n=3).

An additional experiment is obtained with SCE5-HtPlg (4 µg/g BW) pre labelled with anti-6x His tag AF488 antibody (Penta His Alexa-488, Qiagen). The accumulation of the SCE5-HtPlg at the site of the thrombus could then be visualized in the FITC fluorescent channel of the intravital microscope. Snapshots were taken in DIC, FITC

and TRITC channel every 2.5 minutes from 0 to 20 min post injection then every five minutes up to 1 hour post injection.

Lung embolism model

Emboli were induced and fluorescently stained by IV injection (5 µL/g BW) of a mixture of Innovin (5% (v/v) from reconstitution prepared according to manufacturer's instruction) and fibrinogen (10 µg/mL, Sigma) pre-labelled with Cy7-NHS dye (Lumiprobe) at 1:15 molar ratio. This model is similar to thromboplastin induced lung embolism, widely described in the haematology literature[6, 7] and more recently combined with co-injection of fibrinogen labelled with a near-infrared fluorophore to enable quantification of fibrin deposition by fluorescent analysis of the whole lung[8]. 10 minutes after the induction of the prothrombotic mixture, 4 groups of drug were injected intravenously; urokinase at 500 U/g BW, SCE5-HtPlg at 4 µg/g BW, Mut-scFv-HtPlg at 4 µg/g BW and saline (n=3). Mice were killed 50 minutes after the treatment administration and perfused with saline. Lungs, heart, liver, kidney, spleen are harvested and scanned with the Odyssey imaging system in the 700 channel to visualise the organs and in the 800 channel to measure the fluorescence emitted from the near infrared stained emboli. For each animal, signal within the lung was rationalized to signal within the kidney. Results were presented as mean values of fluorescence signal ratio ± SEM (n=3).

Statistical analysis

All results are expressed as mean values ± SEM. Statistical analysis was performed with GraphPad Prism V6 (GraphPad Software, San Diego, CA, USA). Multiple groups (Flow cytometry, tail bleeding, fibrinogen level in plasma, hemoglobin and albumin

levels in brain and intestine and thrombus degradation in both *in vivo* models) were compared with one-way ANOVA and Tukey post-tests. Parameters from *in vitro* fibrinolysis assay of SCE5-HtPlg and Mut-scFv-HtPlg groups were compared with unpaired t tests. A difference of $p<0.05$ was considered significant. Figures 1, 2, 5b and S1 are representative observations with no statistical analysis.

References

1. Nagai N, Demarsin E, Van Hoef B, Wouters S, Cingolani D, Laroche Y, Collen D. Recombinant human microplasmin: Production and potential therapeutic properties. *Journal of thrombosis and haemostasis : JTH*. 2003;1:307-313
2. Schwarz M, Rottgen P, Takada Y, Le Gall F, Knackmuss S, Bassler N, Buttner C, Little M, Bode C, Peter K. Single-chain antibodies for the conformation-specific blockade of activated platelet integrin alphaiibbeta3 designed by subtractive selection from naive human phage libraries. *FASEB journal : official publication of the Federation of American Societies for Experimental Biology*. 2004;18:1704-1706
3. Niego B, Freeman R, Puschmann TB, Turnley AM, Medcalf RL. T-pa-specific modulation of a human blood-brain barrier model involves plasmin-mediated activation of the rho kinase pathway in astrocytes. *Blood*. 2012;119:4752-4761
4. Niego B, Medcalf RL. Improved method for the preparation of a human cell-based, contact model of the blood-brain barrier. *Journal of visualized experiments : JoVE*. 2013:e50934
5. Bonnard T, Hagemeyer CE. Ferric chloride-induced thrombosis mouse model on carotid artery and mesentery vessel. *Journal of visualized experiments : JoVE*. 2015:e52838
6. Weiss EJ, Hamilton JR, Lease KE, Coughlin SR. Protection against thrombosis in mice lacking par3. *Blood*. 2002;100:3240-3244
7. Leon C, Freund M, Ravanat C, Baurand A, Cazenave JP, Gachet C. Key role of the p2y(1) receptor in tissue factor-induced thrombin-dependent acute thromboembolism: Studies in p2y(1)-knockout mice and mice treated with a p2y(1) antagonist. *Circulation*. 2001;103:718-723
8. Lin KY, Kwong GA, Warren AD, Wood DK, Bhatia SN. Nanoparticles that sense thrombin activity as synthetic urinary biomarkers of thrombosis. *ACS nano*. 2013;7:9001-9009

Figure S1

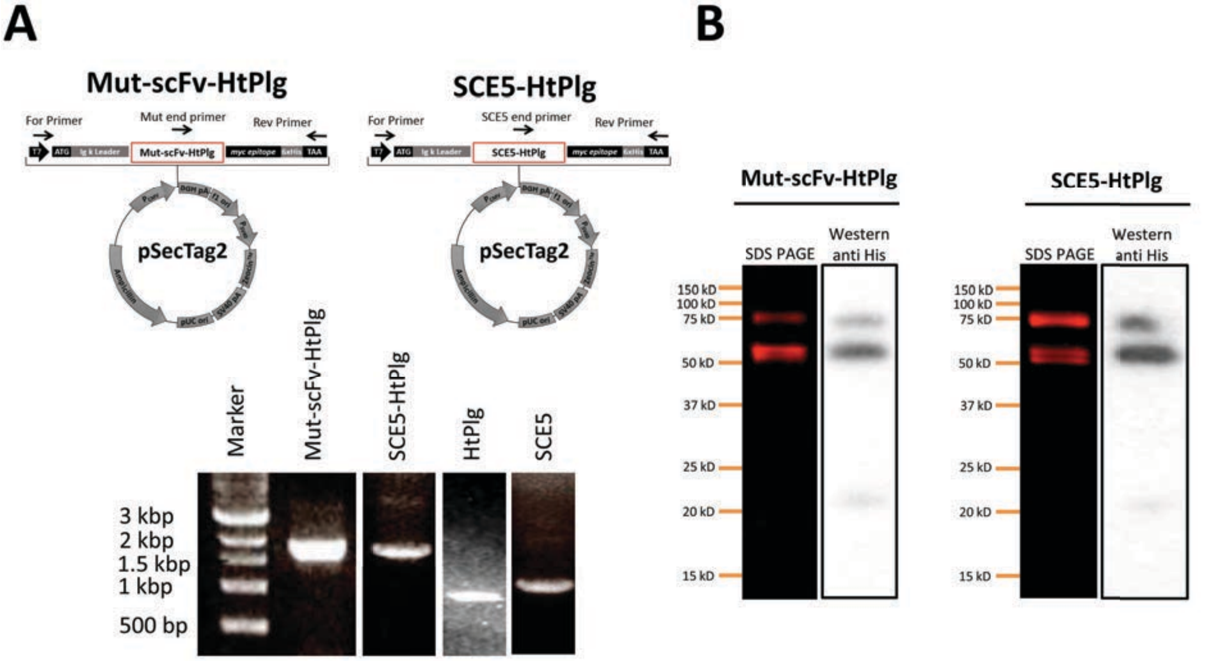

A. Vector map of the non-targeted human thrombin cleavable microplasminogen plasmid (Mut-scFv-HtPlg) and the human thrombin cleavable microplasminogen targeted toward activated GPIIb/IIIa (SCE5-HtPlg). Electrophoresis with 0.8% agarose gel. DNA fragments were digested using restriction enzymes NotI and Xho. Mut-scFv-HtPlg (~1.8 kbp), SCE5-HtPlg (~1.8 kbp) and HtPlg (~0.8 kbp) only after polymerase chain reaction amplification. The undigested (uncut) pSectag vector (negative control) containing the SCE5 single-chain antibody runs at 1 kbp.

B. 12 % SDS-PAGE and Western blot analysis using a horseradish peroxidase coupled to anti-6X His-tag antibody of the Mut-scFv-HtPlg and SCE5-HtPlg.

Figure S2

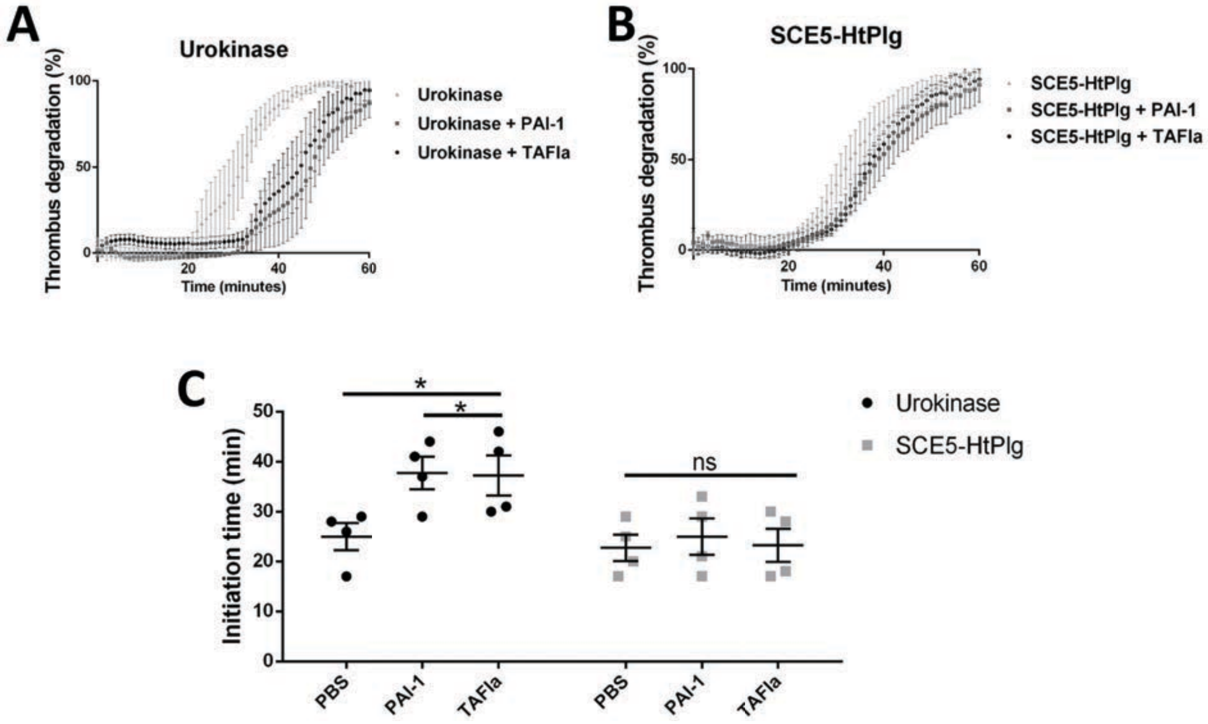

The effect of PAI-1 and TAFIa on the fibrinolytic capacities of Urokinase and of SCE5-HtPlg were tested *in vitro* on thrombi formed in halo shape at the bottom of 96 well plates. Urokinase at 200 U/mL (**A**) and SCE5-HtPlg at 0.2 mg/mL (**B**) was tested in several conditions: (i) incubated 20 minutes in saline and added to blood clots obtained from whole blood, (ii) incubated 20 minutes with 6 nM of PAI-1 and added to blood clots obtained from whole blood, (iii) incubated 20 minutes in saline and added to blood clots obtained from whole blood supplemented with TAFIa (20 nM). The degradation of the thrombi was monitored over 1 hour at 37°C by spectrophotometry from the absorbance of the blood covering progressively the center of the well. Mean thrombus degradation ± SEM is plotted over time (n=4). **C.** Mean thrombus degradation initiation time is presented as mean value ±SEM (n=4, *p<0.05, ns: non-significant).

Figure S3

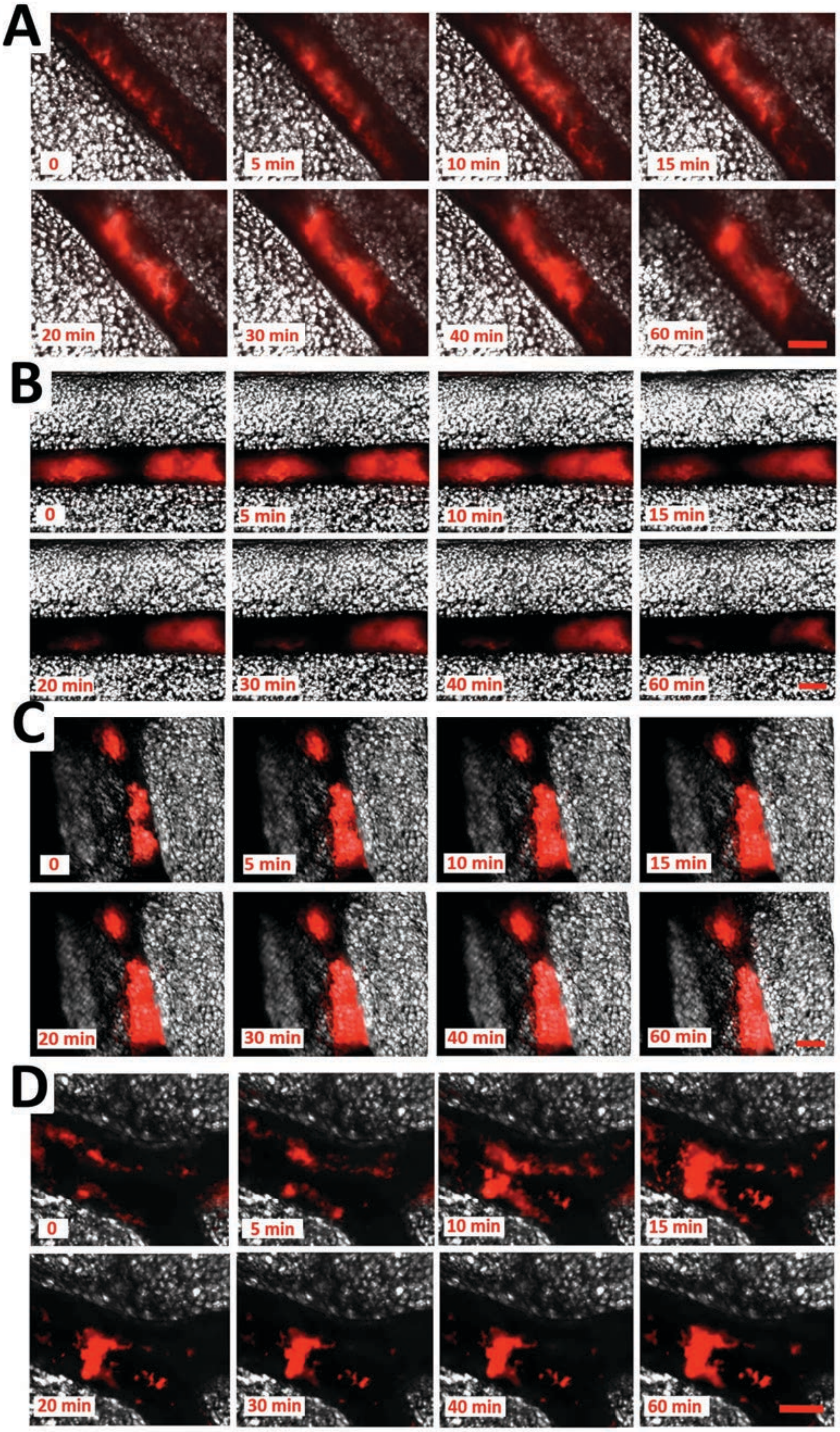
A
0
5 min
10 min
15 min
20 min
30 min
40 min
60 min
B
0
5 min
10 min
15 min
20 min
30 min
40 min
60 min
C
0
5 min
10 min
15 min
20 min
30 min
40 min
60 min
D
0
5 min
10 min
15 min
20 min
30 min
40 min
60 min

Intravital microscopy observations on a vein with a ferric chloride induced thrombus after intravenous injection of SCE5-HtPlg (4 µg/g BW) (**A**), MutMA2-HtPlg (4 µg/g BW) (**B**), SCE5 (1.7 µg/g BW) (**C**) or PBS (**D**). The thrombus is labelled with Rhodamine B. Snapshots were taken in DIC and TRITC channel every 2.5 minutes from 0 to 20 min post injection then every five minutes up to 1 hour post injection. An overlay of the 2 channels at representative time points are presented. Scale bar 200 µm.
